# Isolated Fe-Co heteronuclear diatomic sites as efficient bifunctional catalysts for high-performance lithium-sulfur batteries

**DOI:** 10.1038/s41467-022-35736-x

**Published:** 2023-01-18

**Authors:** Xun Sun, Yue Qiu, Bo Jiang, Zhaoyu Chen, Chenghao Zhao, Hao Zhou, Li Yang, Lishuang Fan, Yu Zhang, Naiqing Zhang

**Affiliations:** 1 State Key Laboratory of Urban Water Resource and Environment, School of Chemistry and Chemical Engineering, Harbin Institute of Technology, Harbin, 150001 China; 2 Space Environment Simulation Research Infrastructure, Harbin Institute of Technology, Harbin, 150006 China; 3 School of Energy Science and Engineering, Harbin Institute of Technology, Harbin, 150001 China

**Keywords:** Batteries, Batteries, Batteries

## Abstract

The slow redox kinetics of polysulfides and the difficulties in decomposition of Li_2_S during the charge and discharge processes are two serious obstacles to the practical application of lithium-sulfur batteries. Herein, we construct the Fe-Co diatomic catalytic materials supported by hollow carbon spheres to achieve high-efficiency catalysis for the conversion of polysulfides and the decomposition of Li_2_S simultaneously. The Fe atom center is beneficial to accelerate the discharge reaction process, and the Co atom center is favorable for charging process. Theoretical calculations combined with experiments reveal that this excellent bifunctional catalytic activity originates from the diatomic synergy between Fe and Co atom. As a result, the assembled cells exhibit the high rate performance (the discharge specific capacity achieves 688 mAh g^−1^ at 5 C) and the excellent cycle stability (the capacity decay rate is 0.018% for 1000 cycles at 1 C).

## Abstract (web summary)

The slow redox kinetics of polysulfides and the difficulties in decomposition of Li_2_S are two serious obstacles to lithium-sulfur batteries. Here, the authors report an isolated Fe-Co heteronuclear diatomic catalyst to achieve high efficiency bifunctional catalysis for lithium-sulfur batteries.

## Introduction

Lithium-sulfur (Li-S) batteries are considered as one of the most promising next-generation energy-storage systems due to its high theoretical capacity, abundant resources and environmental friendliness^1^. Unfortunately, due to the insulation of elemental sulfur, serious shuttle effect of polysulfides (LiPSs), high reaction energy barrier and slow redox reaction kinetics, the actual capacity and long-term stability of Li-S batteries are restricted, which hinders the commercial progress of Li-S batteries^2–4^.

Since the development of Li-S batteries, researchers have made great efforts to explore different sulfur cathode host materials, and made a lot of gratifying progress. Various carbon-based materials and a series of polar adsorption materials have been used to prepare composite cathodes with sulfur due to the limitation of polysulfides^5–7^. In addition, researchers also found that accelerating the conversion rate of LiPSs to reduce the possibility of LiPSs shuttle is a proactive strategy to improve the performance of Li-S batteries. Therefore, many polar catalyst materials^8–11^ (MoS_2_, Co_4_N, FeC) and heterojunction materials^12–15^ (VO_2_-VN, TiO_2_-TiN, MoN-VN) are widely used in Li-S batteries and have achieved excellent electrochemical performance. However, the traditional metal based heterogeneous catalyst materials cannot fully expose their active sites, resulting in a low atomic utilization and limited catalytic effect.

Reducing catalyst size is a recognized and effective strategy to improve catalytic activity. Recently, single metal atomic catalysts (SACs) have been widely used as novel and efficient catalysts in ORR, NRR and other electrocatalysis fields with nearly 100% atomic utilization, high mass activity and unprecedented catalytic activity, and show enormous application potential^16–19^. Niu and co-workers reported that graphene supported Ni single atom catalyst with M-N-C structure has excellent catalytic effect on the reduction of LiPSs to Li_2_S^20–23^. Xie et al.^24^ compared different transition metal SACs (Fe, Co and Ni) and found that Fe-SACs have the strongest adsorption capacity for LiPSs. In addition, adjusting the species of coordination elements and coordination number of metal atoms can also improve the catalytic capacity of SACs^25–27^. Our previous work also demonstrated that the unsaturated coordination Fe-N_2_ site has higher catalytic activity than Fe-N_4_ site^28^. However, unlike other electrocatalytic reactions, the charging and discharging process of Li-S batteries is a reversible redox process. The formation and decomposition of Li_2_S are two reverse oxidation and reduction processes. Introducing an additional metal center into SACs to form a double active sites is a promising strategy to solve the above problems^29–31^. The double active sites are not only a simple doubling of a single atom, they have different catalytic characteristics, and there may be a synergistic effect to break through the theoretical limit of SACs.

Herein, we accurately synthesize the diatomic catalyst (DACs) with Fe-Co dual sites anchored on hollow carbon spheres (Fe-Co DACs) through a two-step solvent impregnation method, which are used as the sulfur host material for the cathode of Li-S batteries. The isolated Fe-Co pairs are clearly observed by aberration-corrected high-angle annular dark-field STEM (HAADF-STEM), and the (Fe-Co-N_6_) structure is also demonstrated by synchrotron-radiation X-ray absorption fine structure spectroscopy (XAFS), indicating that Fe-Co DACs are successfully synthesized and inherit the advantages of fully exposing the active center and maximum atomic utilization of SACs. As a result, the diatomic catalyst show excellent bifunctional catalytic effect during charge and discharge process. In addition, DFT calculation demonstrates that the formation of Fe-Co atomic bond leads to charge redistribution between two atoms, which promotes the adsorption of LiPSs, significantly enhances the adsorption ability and improves the catalytic effect. The DACs cathodes exhibit the high specific discharge capacity of 1001 mAh g^−1^ and excellent cycle stability with a low decay rate per cycle of 0.018% at 1 C for 1000 cycles.

## Results

### Material synthesis and characterization

**Fig. 1:**
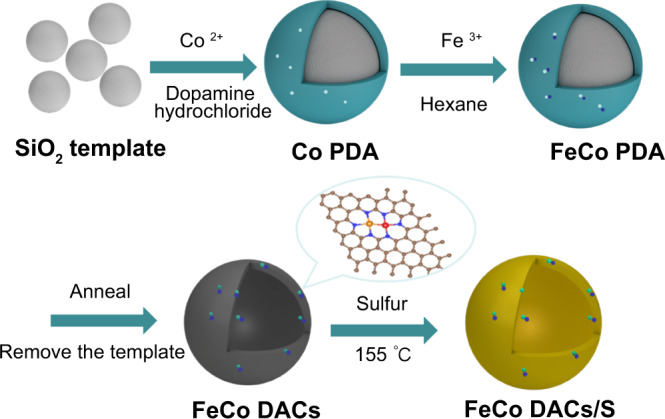
Synthesis scheme. Synthesis scheme of Fe-Co DACs and Fe-Co DACs/S.

**Fig. 2:**
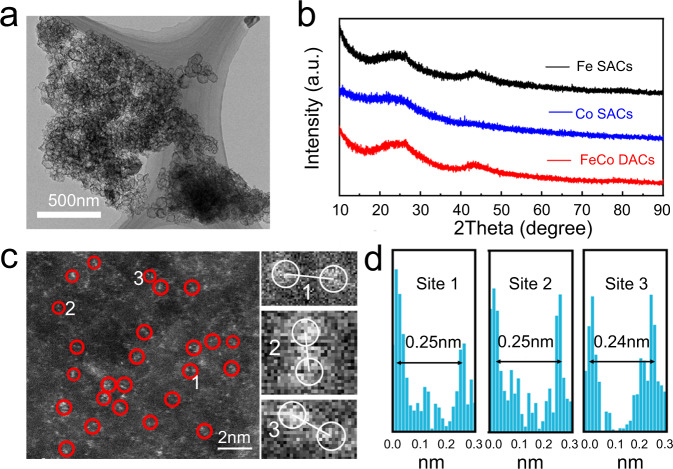
TEM and XRD characterization of Fe-Co DACs. **a** TEM image of Fe-Co DACs. **b** XRD patterns of Fe-CoDACs and Fe(Co) SACs. **c** Aberration-corrected HAADF-STEM image of Fe-Co DACs and some Fe-Co diatomic sites are highlighted by red circles (The red circles 1,2 and 3 are partially enlarged on the right). **d** Intensity profiles of the three sites in c.

The Fe-Co DACs were synthesized by a two-step impregnation method in dual-solvent, as shown in Fig. 1. Firstly, SiO_2_ was used as the template, and dopamine hydrochloride self-polymerized in alkaline condition to form polydopamine (PDA) coating on its surface. At the same time, appropriate Co ions were added to adsorb on the surface of PDA to obtain Co-PDA. Then, the synthesized Co-PDA was ultrasonically dispersed into n-hexane and FeNO_3_ solution was added. The Co site was used as the chemical adsorption site to adsorb polar Fe species in the solution. Finally, FeCoN_6_ supported by nitrogen-doped hollow carbon spheres catalyst (Fe-Co DACs) were obtained after annealing in NH_3_ and removing the SiO_2_ template. The transmission electron microscopy (TEM) images as shown in the Fig. 2a demonstrate that the Fe-Co DACs are hollow spheres of about 50 nm and uniformly distributed. In addition, no agglomeration of nano-particles or lattice stripes are found in the high-resolution transmission image, indicating that the metal atoms did not agglomerate into alloy nano-particles or metal-based compound particles. The N_2_ adsorption/desorption isotherm (BET) in Supplementary Fig. 1 shows that the DACs with high specific surface area of 814.5 m^2^ g^−1^. Supplementary Fig. 2 also demonstrates that Co SACs and Fe SACs have similar specific surface areas as DACs. This hollow structure with high specific surface area not only is conducive to the high dispersion of metal atoms, but also can improve the diffusion of lithium ions and alleviate the volume expansion during the cycle. Supplementary Fig. 3 shows the Raman spectra of DACs and SACs. There are two obvious characteristic peaks at about 1335 cm^−1^ and 1580 cm^−1^, which correspond to the D peak and G peak of carbon materials respectively.

Figure 2b shows the XRD patterns of SACs and DACs, the XRD patterns of the three materials are very similar. The wide diffraction peaks at 26 and 44 degrees correspond to the characteristic diffraction peaks of (002) and (100) of graphitized carbon materials, respectively^32^. No other distinct characteristic peaks exist, which also indicates that the metal atoms are highly dispersed and do not aggregate into nanoparticles. The HAADF-STEM image in Fig. 2c was also used to observe the distribution of metal atoms in Fe-Co DACs. The bright dots represent metal atoms and are marked by red circles. It can be clearly seen that most metal atoms exist in pairs, indicating that diatomic catalysts have been successfully synthesized. In addition, as shown in Fig. 2d the distance between the two atoms is measured to be about 2.5 nm. The isolated atomically dispersed Fe SACs and Co SACs are also demonstrated by HAADF-STEM in Supplementary Fig. 4. The element mapping image in Supplementary Fig. 5 shows that C, N, Fe and Co elements are evenly distributed in the hollow carbon sphere.

**Fig. 3:**
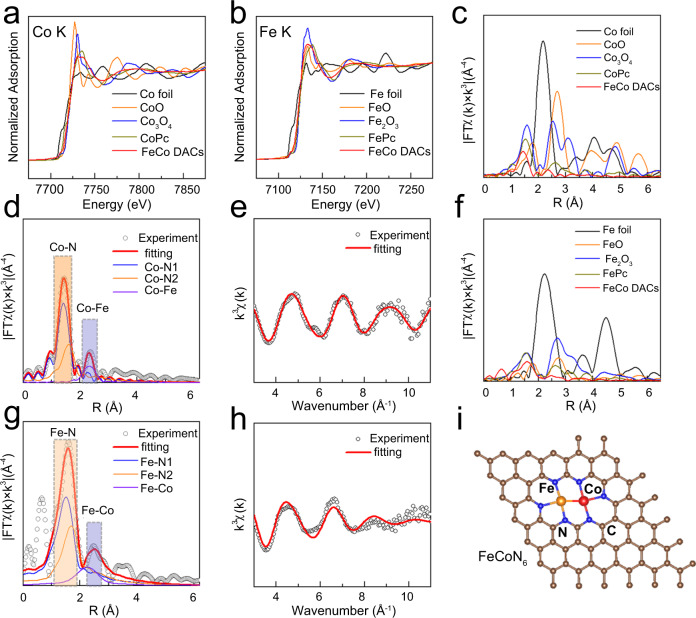
XAS characterizations of the Fe-Co DACs. **a**, **b** Fe and Co K-edge XANES spectra. **c** Fourier transformation (FT)-EXAFS spectra of Co. **d** EXAFS fitting curve of Co in Fe-Co DACs. **e** K-edge k-space experimental EXAFS spectra and fitting curves of Co. **f** Fourier transformation (FT)-EXAFS spectra of Fe. **g** EXAFS fitting curve of Fe in Fe-Co DACs. **h** K-edge k-space experimental EXAFS spectra and fitting curves of Fe. **i** The proposed model of Fe-Co DACs sites.

X-ray photoelectron spectroscopy (XPS) was used to analyze the surface structure and chemical composition of the SACs and DACs. As shown in Supplementary Fig. 6, there are four characteristic peaks in the N 1 *s* spectrum, the peaks at 398.1 eV, 400.7 eV and 401.5 eV can be assigned to pyridine N, pyrrolic N and graphitized N, respectively. The peak at 400.3 eV corresponds to Fe(Co)-N^33–35^. Abundant N-doped sites can also provide additional polar sites for LiPSs adsorption and inhibit the shuttle effect. The Fe 2*p* and Co 2*p* spectral are also shown in Supplementary Fig. 7. The atoms structure and coordination information of DACs were obtained by further analysis of the Fe and Co K-edge XANES (X-ray absorption near-edge structure) and EXAFS (X-ray absorption fine structure measurements) characterization of the samples^36^. Figure 3a shows the Co K-edge XANES spectra and comparative analysis with the test results of Co foil, CoO, CoPc and Co_3_O_4_. The pre-edge of Co K-edge in DACs is located between those of the Co foil and CoO, indicating that the valence state of Co in DACs is between 0 and +2. The XANES spectra of Fe K-edge in Fig. 3b also demonstrates the similar results, and the valence state of Fe is also between 0 and +3. Figure 3c and f shows the Fourier transform of EXAFS spectra of Co (Fe) in DACs and their corresponding compounds. It can be clearly seen that a main characteristic peak and a sub-strong characteristic peak are located at about 1.4 Å and 2.4 Å respectively in the Co spectrum of DACs (Fig. 3c). Combined with the spectra analysis of control samples, it can be concluded that the two characteristic peaks correspond to the Co-N and Co-Fe coordination in the first shell respectively. The k-edge EXAFS fitting curve and fitting results of Co are shown in Fig. 2d, e and Supplementary Table 1. The coordination numbers of Co-N and Co-Fe are 3.1 and 0.8, indicating that most Co atoms combine with adjacent Fe atom to form a dimer structure, which is consistent with the HAADF-STEM images results. On the other hand, EXAFS analysis and fitting results of Fe k-edge in DACs also indicate that the Fe atom mainly bonds with neighboring Co atom and three surrounding N atoms (Fig. 3f–h and Supplementary Table 2). The coordination numbers of Fe-N and Fe-Co are 3.1 and 0.7. In addition, to further verify the coordination structure of Fe-Co DACs, we also used the homonuclear structure of Fe-Fe and Co-Co dual sites to fit the EXAFS results of DACs (Supplementary Fig. 8 and Table 3–4). Obviously, Fe-Co coordination structure has better fit quality and lower R-factor^30^. All the above results demonstrated that the FeCo DACs with N_3_Fe-CoN_3_ coordination structure were successfully synthesized (Fig. 3i). The unique electronic structure and coordination environment may significantly enhance the adsorption and catalytic effect. Inductively coupled plasma atomic emission spectroscopy (ICP-AES) also reveals that the Fe and Co metal contents in FeCo-DACs are 1.08 wt% and 1.03 wt%, respectively (Supplementary Table 5).

### Electrochemical performance test and analysis

**Fig. 4:**
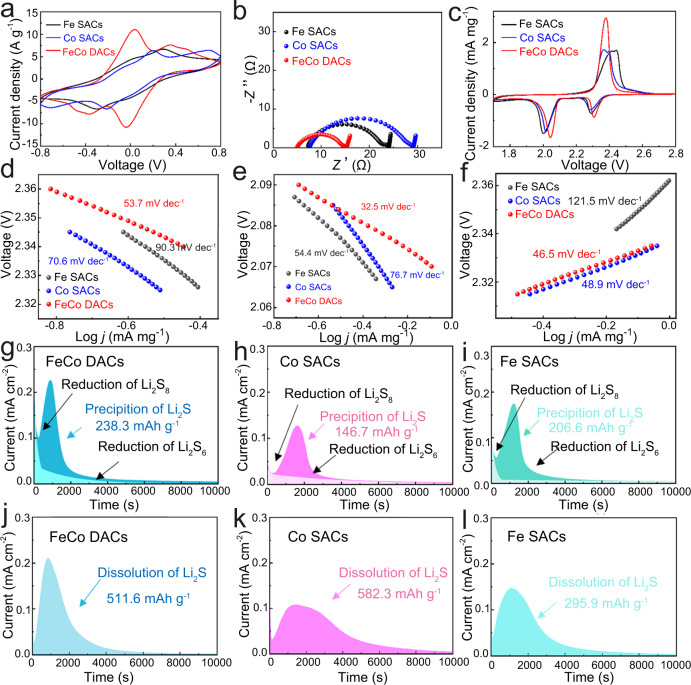
Bifunctional catalytic performance test. **a** CV curves of symmetric batteries at a scan rate of 5 mV s^−1^. **b** Nyquist polts of symmetric batteries. **c** CV curves different cathode at 0.1 mV s^−1^. **d-f** Corresponding Tafel plots of the CV curves. Potentiostatic nucleation curves of Li_2_S with (**g**) Fe-Co DACs, (**h**) Co SACs and (**i**) Fe SACs. The dissolution profiles of Li_2_S with (**j**) Fe-Co DACs, (**k**) Co SACs and (**l**) Fe SACs.

A series of electrochemical performance tests were taken to evaluate the catalytic activity of Fe-Co DACS and SACs. DACs or SACs materials were used as electrodes, and Li_2_S_6_ electrolyte was filled between two same electrodes to prepare symmetrical cells, which were used to analyze the redox reaction kinetics of polysulfides. The CV test is carried out in the voltage range of − 0.8 V ~0.8 V, and the scanning speed is 5 mV/s. As shown in the Fig. 4a, the symmetrical battery with DACs electrode exhibits higher peak current than SACs electrodes, indicating faster redox reaction kinetics, which also proves that DACs have better catalytic effect on the redox process of polysulfide^37,38^. This more efficient catalytic effect of DACs is further demonstrated by electrochemical impedance spectroscopy (EIS) in Fig. 4b. All the EIS spectra are composed of an arc in the high-frequency region and straight line in the low-frequency region, which correspond to the charge transfer process and diffusion process, respectively^39^. DACs electrodes have smaller arc corresponding to smaller charge transfer resistance (R_ct_). The charging and discharging process of Li-S batteries cathode is accompanied by a series of complex solid-liquid-solid conversion process. The DACs and SACs were combined with sulfur as a cathode by melting diffusion method, and Li-S batteries were assembled. As illustrated in Supplementary Figs. 9–10, the content of S is 79.6% and evenly distributed, indicating that it can be captured by the carbon spheres. Then, cyclic voltammetry was used to study the battery reaction process with a voltage range of 1.7–2.8 V with a scanning speed of 0.1 mV /s (Fig. 4c). The CV result shows two obvious reduction peaks and one oxidation peak, corresponding to S_8_^2−^ → S_4_^2−^, S_4_^2−^ → Li_2_S_2_/Li_2_S and Li_2_S_2_/Li_2_S → S_8_ reactions, respectively. Interestingly, the oxidation peak potential of Co SACs is significantly lower than that of Fe SACs, indicating that CoN_4_ is more favorable to the charging process of Li-S batteries than FeN_4_, while Fe SACs has a higher reduction peak current, indicating that it is favorable to the discharge reaction process. Compared with SACs, the oxidation peak of DACs shifts significantly to the direction of negative voltage, while the reduction peak shifts to the direction of positive voltage, showing smaller electrochemical polarization and excellent reversibility. Meanwhile, DACs cathode also shows a higher peak current density, which also indicates that DACs can significantly accelerate the redox reaction kinetics rate of polysulfides. The CV curves in Supplementary Fig. 11 also indicate that DACs have better electrochemical performance than Fe/Co SACs mixtures and hollow carbon spheres. The Slope of Tafel in Fig. 4d–f was further calculated to analyze the catalytic activity of different reaction processes. And the fitting results of the Tafel slope are shown in Supplementary Fig. 12. Remarkably, in comparison with the Fe-N_4_ SACs, Co-N_4_ SACs are significantly more favorable for oxidation reactions (charging processes). However, in the reaction step of S_4_^2−^ → Li_2_S_2_/Li_2_S, Fe SACs show a lower Tafel slope than Co SACs, indicating that Fe single atom is beneficial for the discharge process. The slope of Tafel in the oxidation and reduction process of DACs cathode are lower than that of SACs cathode, demonstrating that Fe-Co DACs catalyst can achieve efficient dual-function catalytic effect on the oxidation and reduction reaction process of Li-S batteries simultaneously. This may be attributed to the synergistic catalytic effect between Fe and Co atoms.

In general, the nucleation and decomposition of Li_2_S are accompanied by liquid-solid and solid-liquid transitions in the cycle of Li-S batteries, which have the highest reaction energy barrier and are the rate-limiting steps of charging and discharging processes, respectively. The potentiostatic discharge and galvanostatic charge experiments were designed to investigate the nucleation and the growth of Li_2_S in discharging process and Li_2_S decomposition in charging process, respectively^40,41^. As shown in Fig. 4g–i, the DACs electrodes show the highest nucleation current and the earliest nucleation time. Moreover, the capacity of Li_2_S precipitation on DACs is much larger than those on Fe SACs and Co SACs, indicating that the Fe-CoN_6_ diatomic active sites could can significantly promote the nucleation and growth of Li_2_S. However, the Li_2_S dissolution capacity of DACs and Co SACs are much higher than Fe SACs, which reveals that the DACs and Co SACs can effectively promote Li_2_S dissolution in the charge process. Fe/Co mixed SACs samples were also used to assemble cells for nucleation and dissolution experiments to evaluate the synergistic effect of Co and Fe atoms in DACs. As shown in Supplementary Fig. 13, the cells with Fe-Co DACs show higher nucleation and dissolution capacity than the cells with Fe-SAC/Co-SAC mixed electrode. These results further prove that the dual-function catalytic activity of Fe-Co DACs can facilitate the rapid charging and discharging process at the same time, and improve the utilization rate of active substances.

### Chemical adsorption interaction of polysulfides and in situ characterization

**Fig. 5:**
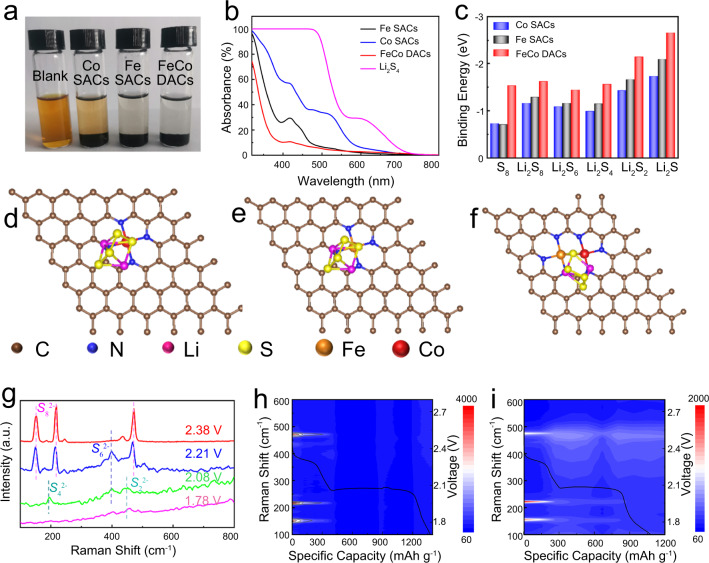
Adsorption performance test and in situ characterization. **a** Optical photograph of visualized adsorption tests of Li_2_S_4_ with different materials. **b** Corresponding UV–vis spectrums after 12 h. **c** Binding energies between the LiPSs and different materials. **d–f** Adsorption model of Li_2_S_4_ on different materials. **g** In-situ Raman spectra of Fe-Co DACs/S at differenent voltages. **h**, **i** In situ Raman contour plots and corresponding discharging curves of Fe-Co DACs/S and Co SACs/S cathode.

Severe shuttle effect is the main culprit of capacity attenuation in the cycle of Li-S batteries, so the restriction of polysulfide is the key factor to evaluate the main material of Li-S batteries. Visual adsorption experiments were taken to evaluate the adsorption performance of DACs and SCAs materials. The same weight Fe-Co DACs, Fe SACs and Co SACs were added to Li_2_S_4_ solution and aged for 12 h, as shown in Fig. 5a. Obviously, the color of Li_2_S_4_ solution with DACs is more colorless and transparent than SACs solution, indicating that DACs has a stronger adsorption capacity for LiPSs than SACs. In addition, the color of Li_2_S_4_ solution with Fe SACs was lighter than that with Co SACs, demonstrated that the adsorption capacity of Fe SAC to Li_2_S_4_ was better than that of Co SACs. This result is further supported by the corresponding UV/vis adsorption spectra in Fig. 5b. Density functional theory (DFT) calculations were employed to further investigate the adsorption energy between different LiPSs and DACS and SACs sites. The corresponding binding energy (E_ads_) is calculated, and the more negative E_ads_ means the stronger anchoring effect of LiPSs^42^. As shown in Fig. 5c, compared with SACs, DACs show the strongest adsorption capacity for all kinds of LiPSs, which is beneficial to suppress the shuttle effect and improve the utilization of sulfur species and cycle stability. The adsorption configurations of different materials are shown in Supplementary Fig. 14–16.

In situ Raman spectroscopy was used to further characterize the conversion process of LiPSs and the inhibition of shuttle effect during the discharge process^43^. As shown in Fig. 5g, at 2.38 V, the battery just starts to discharge, and there are three obvious characteristic peaks in the Raman spectrum at 156, 217 and 475 cm^−1^, corresponding to S_8_ and S_8_^2−^. As the reaction continues (2.21 V and 2.08 V), the characteristic peaks of S_8_^2−^ gradually disappear, the characteristic peaks of S_6_ (396 cm^−1^) and S_4_^2−^ (198 cm^−1^) appear, and they are all converted into Li_2_S_2_ (452 cm^−1^) and Li_2_S at the end of the final discharge (1.78 V). In-situ Raman contour plots of Li-S batteries assembled with different cathodes during the initial discharge process are shown in Fig. 5h, i and Supplementary Fig. 17. It is obvious that the Raman signals of various Li_2_S_8_ at the cathode of DACs can hardly be detected after the first discharge plateau. Obviously, the characteristic peak of Li_2_S_8_ generated at the initial discharge stage of DACs cathode disappeares after the end of the first discharge platform, demonstrating that it is completely transformed. In addition, the Raman signals of long-chain LiPSs can hardly be detected after discharge, further confirming that DACs can promote the complete conversion of LiPSs and effectively alleviate shuttle effect.

### Electrochemical energy-storage performances of Li-S batteries

**Fig. 6:**
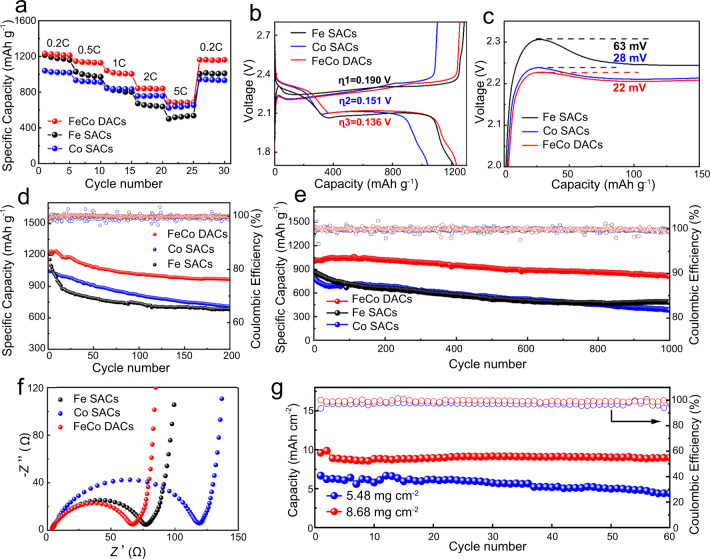
Electrochemical performance of Li-S batteries based on different cathodes. **a** Rate performance at different specific current (1 C corresponds to 1675 mA g^−1^). **b** Corresponding galvanostatic charge-discharge curves at 0.2 C. **c** The first charge voltage profles. **d** Cycle performance at 0.2 C. **e** The long-cycle performance of different cathodes at 1 C. **f** Nyquist polts of different cathodes. **g** Cycle performance of Fe-Co DACs/S cathode with high sulfur loading at 0.1 C.

To evaluate the improvement of DACs on the electrochemical performance of Li-S batteries, coin cells were assembled with different cathodes and galvanostatic charge-discharge tests were taken with different current densities. As expected, the DACs/S cathode exhibits excellent rate capacities of 1233, 1147, 1041, 841and 688 mAh g^−1^ at 0.2, 0.5, 1, 2 and 5 C, respectively, which are significantly better than Fe SACs cathodes and Co SACs cathodes at each current density (Fig. 6a). Figure 6b shows the charge-discharge curves of different cathodes at 0.2 C. The overpotential (η) of DACs/S cathodes is 0.136 V, significantly lower than that of Fe SACs/S cathodes (0.151 V) and Co SACs/S cathodes (0.190 V), which indicate the rapid reaction kinetics of DACs electrocatalyst. As shown in Fig. 6c, both DACs and Co SACs show low overpotentials at the initial charging stage, representing a lower charging energy barrier and promoting the decomposition of lithium sulfide, which is also consistent with the experimental results of lithium sulfide dissolution. The gap between charge and discharge potentials becomes more obvious at high current densities (Supplementary Fig. 18). This also demonstrates the dual-function catalytic activity of DACs during charging and discharging. The DACs cathode provides an initial capacity of 1227 mAh g^−1^ at 0.2 C and the capacity retention rate can reach 79% after 200 cycles, which is higher than that of Fe SACs /S cathodes and Co SACs/S cathodes (Fig. 6d and Supplementary Fig. 19). In addition, as shown in Fig. 6e, the DACs/S cathode also shows excellent discharge performance and cycle stability in long-term cycle stability measurements at higher current density of 1 C. The initial discharge capacity is 1001 mA h^−1^ and the capacity decay rate is 0.018% per cycle of 1000 cycles. By contrast, the capacity decay rates of Fe SACs/S cathode and Co SACs/S cathode are 0.043% and 0.053%, respectively. The excellent cyclic stability is attributed to the high efficiency of bifunctional catalytic activity and the strong anchoring ability to LiPSs. The EIS impedance test results in Fig. 6f also demonstrate that DACs/S has a smaller charge transfer impedance. The SEM characterization of lithium anode after cycling (Supplementary Fig. 20) also shows that compared with SACs cells, the surface of lithium anode of DACs cell is smoother and the corrosion degree is lighter, which is due to the strong catalytic performance of DACs and better inhibition effect on shuttle effect.

The discharge performance of Li-S batteries under high sulfur area loading conditions is also an important index to evaluate their application potential. As shown in Fig. 6g, even when the sulfur loading of DACs /S cathode is increased to 5.48 and 8.68 mg cm^−2^, the cell still achieves high initial capacity of 6.67 and 9.59 mAh cm^−2^ and exhibits good cycling performance. The electrochemical performance of the cell is still excellent under the condition of lean electrolyte (Supplementary Fig. 21). Pouch cell was also assembled for electrochemical performance testing. The optical photo and cycle performance curves of the pouch cell are shown in Supplementary Fig. 22.

### DFT calculation analysis of the origin of catalytic activity

**Fig. 7:**
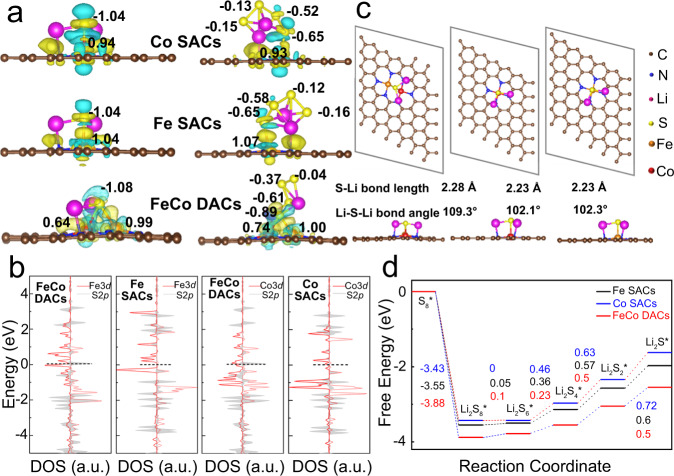
DFT calculations of Fe-Co DACs, Fe SACs and Co SACs catalysts. **a** Charge density difference plot of Li_2_S (Left) and Li_2_S_4_ (Right) adsorbed on different materials. **b** DOS of Li_2_S_4_ adsorbed on Fe-Co DACs, Co SACs and Fe SACs systems. **c** Optimized configurations of Li_2_S anchored on Fe-Co DACs, Co SACs and Fe SAs-NG. **d** Gibbs free energy of sulfur reduction process on Fe-Co DACs, Co SACs and Fe SACs.

DFT calculation was carried out to further analyze the intrinsic mechanism of enhanced catalytic activity of DACs. The decomposition of Li_2_S and the liquid-solid conversion of Li_2_S_4_ to Li_2_S are the rate-limiting steps in the charging and discharging process respectively, which are also the main reasons for the decrease of the utilization rate of active substances and the formation of dead sulfur. Figure 7a displays the charge density difference plot of Li_2_S and Li_2_S_4_ adsorbed on DACs (SACs), respectively. Due to the synergistic effect between Co and Fe in the diatomic active site, the electron transfer between DACs and Li_2_S is 1.08 e^−^, which is higher than SACs. Similarly, according to the Bader charge analysis, the S atoms in Li_2_S_4_ transfer charges to FeCoN_6_ site as high as −0.89, −0.61, −0.37 and −0.04 e^−^, respectively, which are also significantly higher than those of Fe SACs and Co SACs. Therefore, the interaction between diatomic sites is beneficial for accelerating the electron transfer in the kinetic transformation process, reduce the reaction energy barrier, and promote the nucleation and decomposition of Li_2_S. In addition, the density of states (DOS) of Li_2_S_4_ adsorbed on the surface of DACs was also calculated to confirm the strong interaction between the metal 3*d* electron and the S 2*p* electrons. As shown in Fig. 7b, the Li_2_S_4_-DACs adsorption system has a more significant DOS near the Fermi level, indicating the faster electron transfer between FeCoN_6_ sites and Li_2_S_4_, accelerating the conversion of LiPSs. This is attributed to the synergistic effect between Co and Fe. The orbital interaction between Co and Fe redistributes the 3*d* states of DACs, resulting in less localized of 3*d* states and d-orbitals crossing the Fermi level. The decomposition pathways and energy barriers of Li_2_S on different catalyst surfaces were calculated in Supplementary Fig. 23, demonstrated Fe-Co DACs and Co SACs have better catalytic activity for Li_2_S decomposition, which is consistent with the results of Li_2_S dissolution experiment. Figure 7c illustrates the optimized adsorption configuration of Li_2_S on DACs surface. Compared with the SACs systems, Li_2_S adsorbed on the surface of DACs shows longer Li-S bonds and larger Li-S-Li bond angles. In addition, it is generally considered that there are multiple reaction steps and intermediate products (S_8_, Li_2_S_8_, Li_2_S_6_, Li_2_S_4_, Li_2_S_2_, and Li_2_S) during the discharge process of lithium-sulfur batteries, so the Gibbs free energy change (ΔG) for each lithiation step is also calculated in Fig. 7d^44^. Homonuclear Fe-Fe and Co-Co DACs were also used in DFT calculations and show similar results in Supplementary Fig. 24. It can be seen that the free energy of the LiPSs reduction reaction on the surface of DACs is smaller than that of the corresponding SACs surface, which indicates that the discharge process of the DACs/S cathode is more favorable from the perspective of thermodynamics.

## Discussion

In summary, we successfully prepare the hollow carbon sphere supported atomically dispersed Fe-Co DACs with bifunctional catalytic activity as sulfur host materials for Li-S batteries. Electrochemical tests, combined with in situ Raman spectroscopy and theoretical calculations show that this unique dual-sites structure can not only enhance the anchoring ability to LiPSs, but also accelerate the reaction kinetics of LiPSs conversion and Li_2_S decomposition, achieving efficient dual-function catalytic effect during charging and discharging processes simultaneously. Accordingly, the DACs/S cathodes exhibit the high discharge specific capacity and the excellent cycle stability, which include high rate performance (688 mAh g^−1^ at 5 C) and a low capacity decay rate of 0.018% per cycle for 1000 cycle at 1 C. This work contributes an in-depth understanding of the synergy of multi-center catalytic active sites and broadens the application of atomically dispersed catalytic materials in Li-S batteries.

## Methods

### Synthesis of Co-PDA

1 mL ammonia water and 24 mL ethanol were added to 80 mL deionized water and stirred for 30 min. Then 2 mL ethyl orthosilicate(TEOs) was dropped and stirred for 30 min to form SiO_2_ suspension.60 mg cobalt nitrate was added, then 6 mL dopamine hydrochloride solution (0.3 M) was quickly added to the above solution and stirred for another 48 hours, followed by centrifugation and vacuum drying.

### Synthesis of Fe-Co-PDA

100 mg of the Co-PDA precursors were dispersed in 50 mL n-hexane, and ultrasound was performed for 2 h until uniform dispersion. Then 0.2 mL ferric nitrate solution was added drop by drop to the above n-hexane solution, and ultrasound was continued for 2 h, followed by 12 h agitation to ensure full adsorption between the single atomic sites and the iron species. Then centrifuge, wash, and vacuum dry to obtain Fe-Co PDA.

### Synthesis of Fe-Co DACs

The Fe-Co PDA powder was transferred to a tubular furnace and calcined at 900 °C for 2 h in the atmosphere of ammonia. Then the SiO_2_ template was removed with NaOH solution and the residual metal was removed with 0.5 M H_2_SO_4_. Finally, Fe-Co DACs materials were obtained. The control group used the same synthesis method, but the difference is that for the first step of Fe SACs synthesis, cobalt nitrate is not added, only PDA is synthesized. In addition, for the step two the ferric nitrate solution increased to 0.4 mL. Similarly, when synthesizing Co SACs, 120 mg of cobalt nitrate was added in the first step and no iron nitrate was added in the second step.

### Synthesis of Fe-Co DACs /S, Co SACs /S and Fe SACs /S composites

Fe-Co DACs /S cathode were obtained by combining host materials with S by a simple fusion diffusion method. The host material powder was mixed with S in a mass ratio of 2:8 and then ground with a mortar for 1 h. Next, the mixture was transferred to a polytetrafluoroethylene reactor and heated at 155 °C for 12 h to obtain Fe-Co DACs /S cathode materials. Replaced Fe-Co DACs with Co SACs (or Fe SACs) and use the same method as above to get the other cathode materials.

### Characterizations

XRD patterns were recorded at X’Pert PRO diffractometer. The SEM (SU8010), TEM(Tecnai G2 F30) and spherical aberration corrected scanning transmission electron microscopy (STEM, JEM-ARM200F) were used to obtain the morphological of catalyst materials and electrode. Micromeritics ASAP 2020 analyzer was carried to test the specific surface area of DACs and SACs. The X-ray Absorption Spectroscopy of Fe-Co DACs was recorded at Beijing Synchrotron Radiation Facility (BSRF) with the 1W1B beamline. Surface chemical compositions and elemental valence were analyzed with XPS (Thermo Scientific). In situ Raman spectra were tested on a Lab RAM HR800 Raman spectrometer.The wavelength of the incident laser was 532 nm. Uv-2450 UV-visible spectrophotometer was used for the UV test of LiPSs adsorption experiment. The content of metal elements was analyzed by inductively coupled plasma emission spectrometer (ICP, NexION 350X).

#### Electrochemical measurements

Cathode was prepared by pasting the slurry prepared with 80 wt% active material (Fe-Co DACs /S, Co SACs /S and Fe SACs /S), Super P(10 wt%) and PVDF(10 wt%) on the carbon-coated aluminum foil collector plate. After drying, cut into circular pellets for assembly of batteries. And the sulfur areal loading was about 1.2 mg cm^−2^. The Celgard 2400 sheet (16 mm) was as the commercial separator and the lithium plate (15 mm) as an anode to form a 2025 coin cells. All the cells were assembled in a glove box filled with argon gas. Each cell injecte 20 µL electrolyte and the electrolyte was composed with 1 mol/L lithium bis (trifluoromethanesulfonyl) imide (LiTFSI), 2% LiNO_3_ in a solvent of 1, 3-dioxolane (DOL) and dimethoxymethane (DME) (1:1 ratio by volume). For the high sulfur loading cell, the sulfur loading was 5.48 and 8.68 mg cm^−2^, the electrolyte dosage is 75 µL. After static for 12 hours, galvanostatic charge-discharge test was carried out with the voltage range of 1.7 to 2.8 V (Neware, Shenzhen, China). In addition, all electrochemical tests in this work are performed at ambient temperatures (about 25°C).

#### Fabrication of Li-S pouch cell

The composition of the cathode material was prepared by the same method with the coin cell. The slurry was coated on carbon-coated aluminum foil and the sulfur loading is about 3 mg cm^−2^. After drying at 60 °C for 12 h, the coated aluminum foil was cut into 5.5 cm × 6 cm shape and used as cathode. The sulfur loading of one piece cathode was about 100 mg. The thickness of lithium anode and Cu collector are 100 μm and 10 μm, respectively. The Al tab was riveted on the cathode, and Ni tab riveted on the Cu foil current collector. The pouch cell is assembled with two pieces anodes and two pieces cathodes. Two pieces of Li foil were placed on both sides of the Cu foil. Then, we stacked a layer of Celgard 2400 on the surface of the Li foil and stacked the two pieces cathode at the top and bottom of the Celgard 2400 separator, respectively. The electrolyte amount was controlled to achieve E/S ratio of 4.0 µL mg^-1^(800 μL for a 200-mg sulfur load pouch cell). Then, the package was sealed under vacuum. Finally, the as prepared pouch cell showed the size of 6 cm × 7 cm. The assembled pouch cell was tested after standing for 12 h, and the voltage range was the same as that of the coin cell.

#### Li_2_S_6_ symmetric cell measurements

The catalyst material, PVDF and P were uniformly mixed to form a slurry with a weight ratio of 8:1:1. Next, the slurry is applied to the aluminum foil. Next, the slurry is coated on carbon-coated aluminum foil and dried at 60 °C for 12 h. After drying, it is cut into slice with a diameter of 12 mm and the areal mass loading of catalyst materials is about 0.5 mg cm^−2^. The cut electrodes are used as both the cathode and the anode of a symmetrical cell. The electrolyte was 0.2 M Li_2_S_6_ and 1 M LiTFSI solvated in DME/DOL (vol/vol 1:1) with 1 wt% LiNO_3_. The separators of symmetric cells also use the Celgard 2400 separator. The assembled symmetric cells were tested for cyclic voltammetry at a potential range of −0.8 to 0.8 V and a scanning speed of 5 mV s^−1^. The CHI660 workstation was used for testing and there was no IR compensation during testing.

#### Li_2_S precipitation and dissolution experiments

The precipitation and dissolution test cells used the same cathode and separator as the symmetric cells. The difference is that they use lithium metal as anode. In addition, the electrolyte of Li_2_S precipitation and dissolution test was 0.2 M Li_2_S_8_ and 1 M LiTFSI solvated in DME/DOL (vol/vol 1:1) with 1 wt% LiNO_3_. In the process of Li_2_S precipitation test, the prepared cells were first galvanostatically discharged at a current of 0.1 mA to 2.06 V, followed by potentiostatically discharged at 2.05 V. For the Li_2_S dissolution test, the prepared cells were first galvanostatically discharged at a current of 0.1 mA to 1.7 V, followed by potentiostatically charged under 2.4 V.

#### Computational methods

All ab initio calculations used in this work were performed using density functional theory (DFT) methods with the Quantum ESPRESSO package. The 15 × 15 × 1 Monkhorst-Pack k-point grid was used to optimize the equilibrium lattice constant. We use it to construct a 6 × 6 × 1 graphene sheet model with vacuum layer of 15 Å. Then Fe-CON_6_ or Fe(Co)-N_4_ structures are simulated on this base model. All the atoms are allowed to fully relax during the structural optimization process.

#### Reporting summary

Further information on research design is available in the Nature Portfolio Reporting Summary linked to this article.

## Supplementary information

Supplementary Information

## Data Availability

The data that support the findings of this study are available from the corresponding authors upon reasonable request.
