# Supplementary material for: Isolated Fe-Co heteronuclear diatomic sites as efficient bifunctional catalysts for high-performance lithium-sulfur batteries
Source: Nat Commun. 2023 Jan 18;14:291. doi: 10.1038/s41467-022-35736-x (PMC9849388; doi:10.1038/s41467-022-35736-x)
Supplement: Supplementary file 1 — Supplementary Information [file 41467_2022_35736_MOESM1_ESM.pdf]

Supplementary Information

Isolated Fe-Co heteronuclear diatomic sites as efficient bifunctional catalysts for high-performance lithium-sulfur batteries

Xun Sun [a], Yue Qiu [a], Bo Jiang [a], Zhaoyu Chen [b], Chenghao Zhao [a], Hao Zhou [a],Li Yang [a], Lishuang Fan [a], Yu Zhang [*c] , Naiqing Zhang [*a]

[a] State Key Laboratory of Urban Water Resource and Environment, School of Chemistry and Chemical Engineering, Harbin Institute of Technology, Harbin, 150001, China.

[b] Space Environment Simulation Research Infrastructure, Harbin Institute of Technology Harbin 150006, China

[c] School of Energy Science and Engineering, Harbin Institute of Technology, Harbin, 150001, China.

*Corresponding Author

Naiqing Zhang (zhangnq@ hit.edu.cn)

Yu Zhang (zhangchemistry@hit.edu.cn)

School of Chemistry and Chemical Engineering, Harbin Institute of Technology, Harbin, China

Tel: +451 86412153 Fax: +86-451-86412153

Table of contents:

Supplementary Figures 1-23
Supplementary Tables 1-5

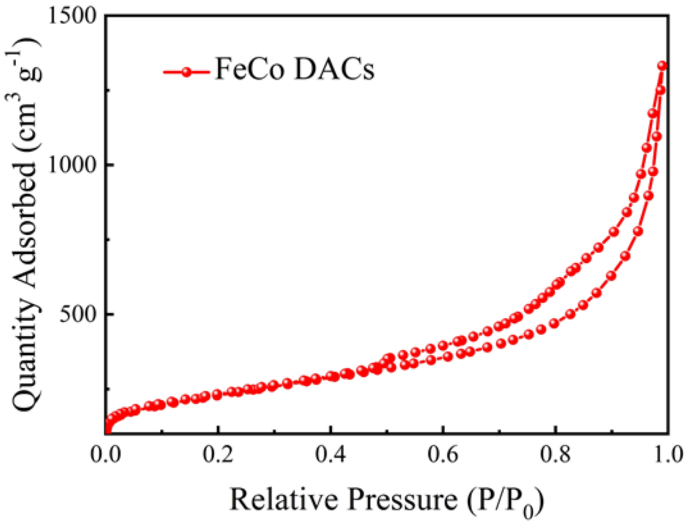

Supplementary Fig. 1 N_2 isothermal adsorption-desorption curves of FeCo DACs.

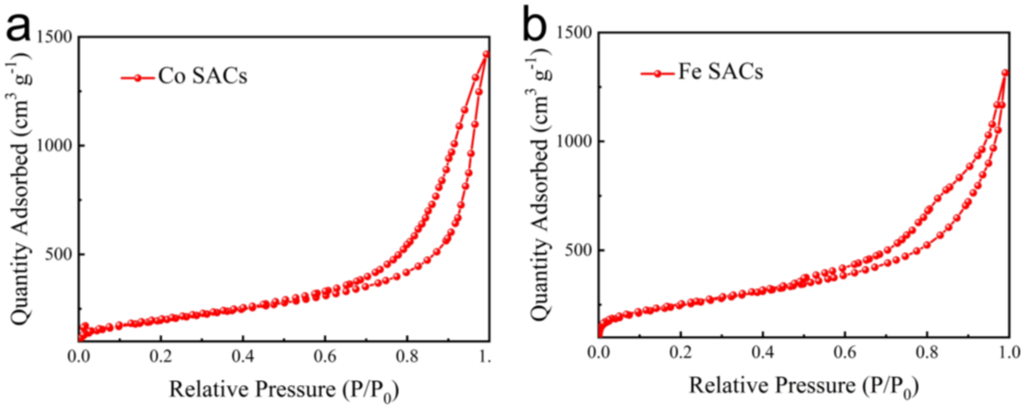

Supplementary Fig. 2 N_2 isothermal adsorption-desorption curves of (a) Co SACs,(b) Fe SACs.

The specific surface areas of Co SACs and Fe SACs are 828.9 and 812.4, respectively.

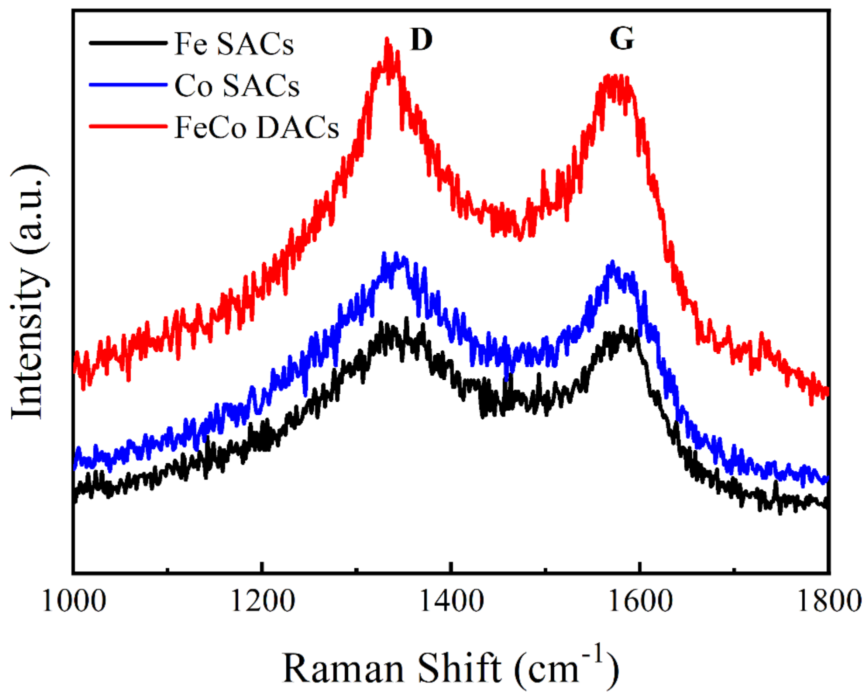

Supplementary Fig. 3 Raman spectra of FeCo DACs, Co SACs and Fe SACs.

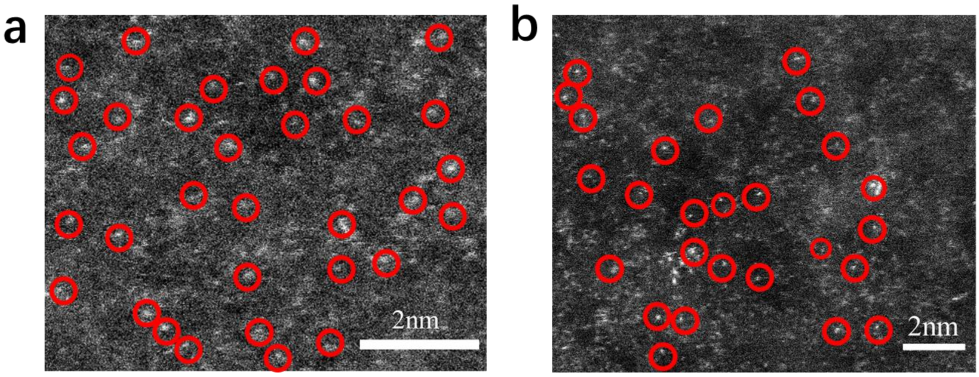

Supplementary Fig. 4 Aberration-corrected HAADF-STEM image of (a) Co SACs and (b) Fe SACs.

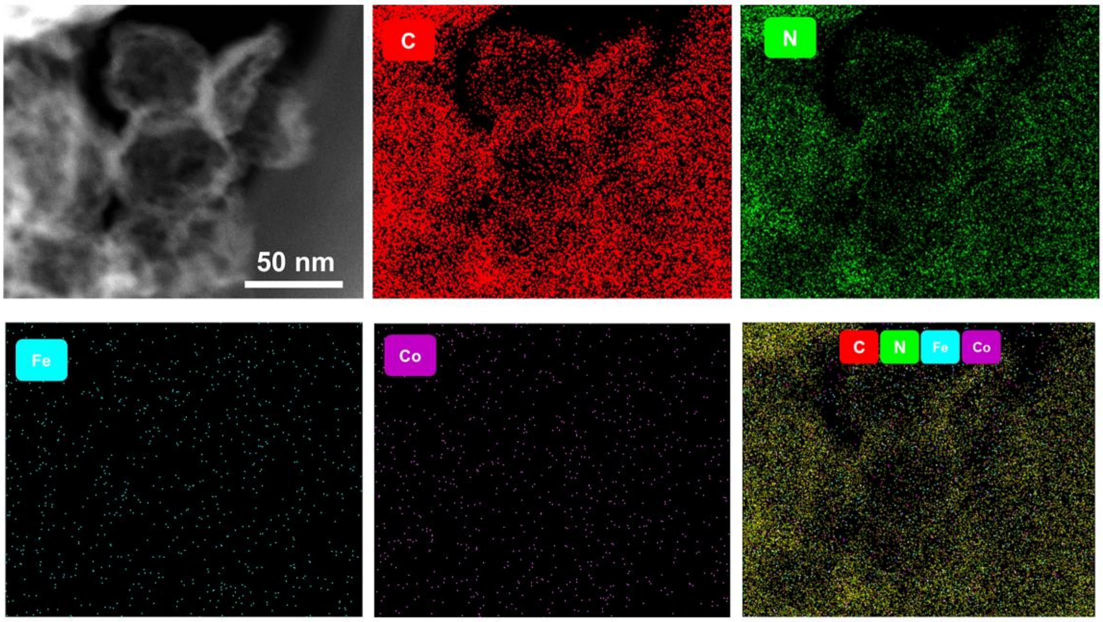

Supplementary Fig. 5 The elemental mapping images of FeCo DACs.

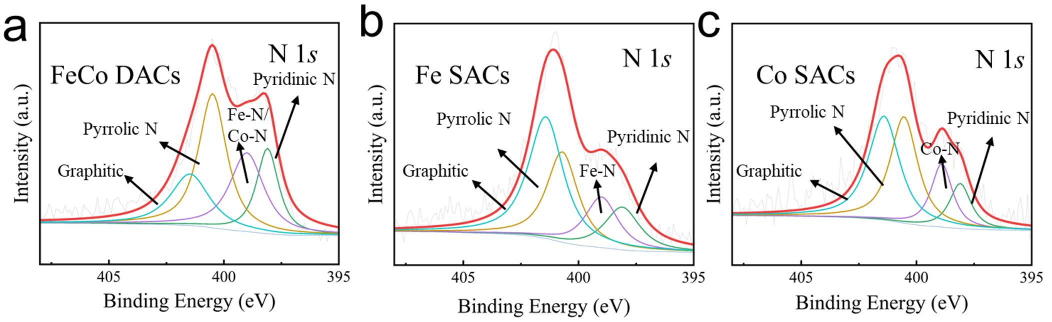

Supplementary Fig. 6 High-resolution N 1s XPS spectra of the (a) FeCo DACs, (b) Fe SACs and (c) Co SACs .

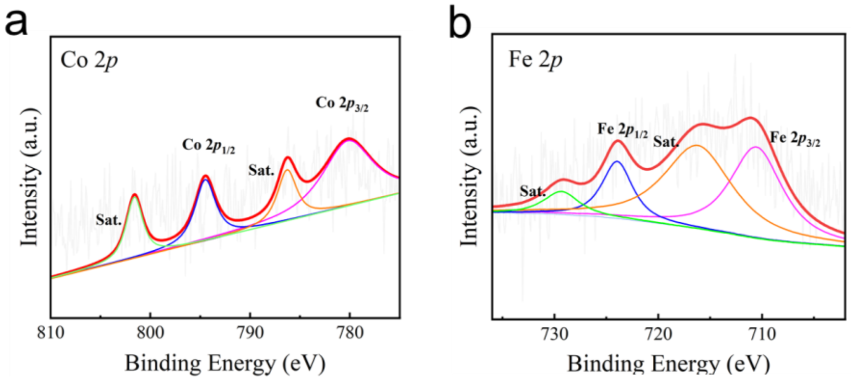

Supplementary Fig. 7 XPS spectra of the FeCo DACs (a) Co 2p (b)Fe 2p.

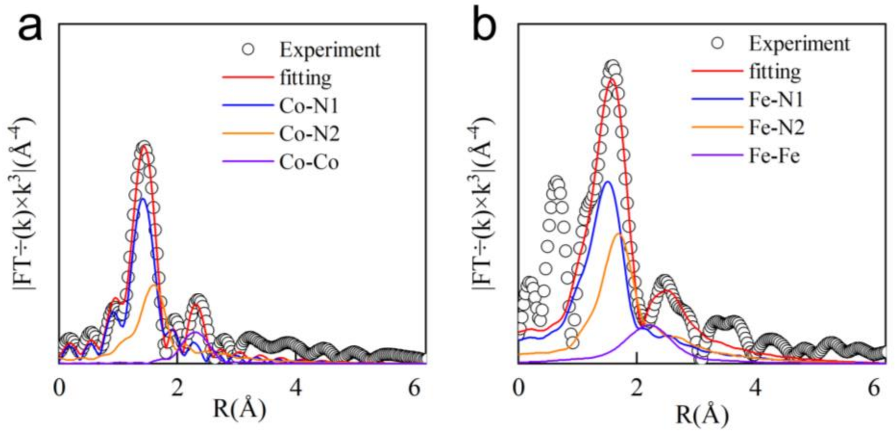

Supplementary Fig. 8 (a) The Co K-edge (b) Fe K-edge EXAFS fitting curves of Fe-Co DACs with Co-Co and Fe-Fe.

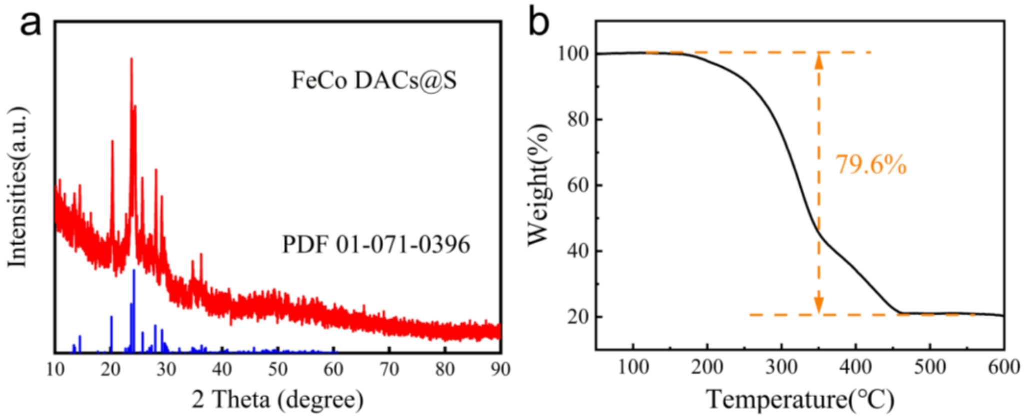

Supplementary Fig. 9 (a) XRD patterns of Fe-CoDACs/S composites. (b) TGA curve of Fe-CoDACs/S composites.

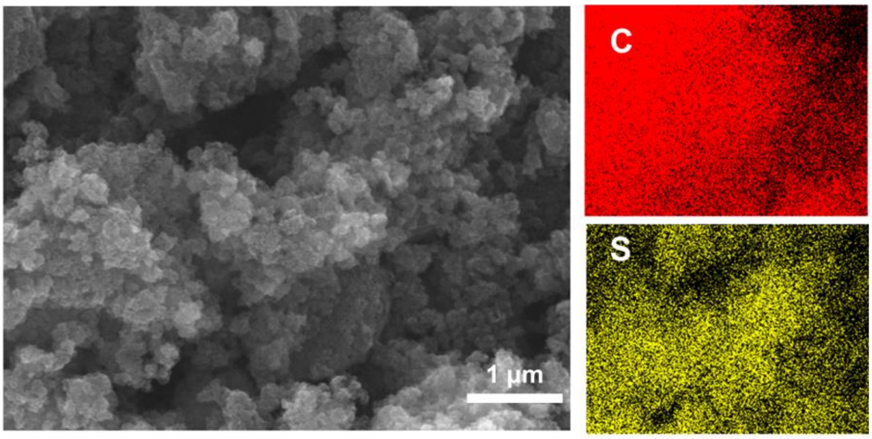

Supplementary Fig. 10 Elemental mapping images of C and S.

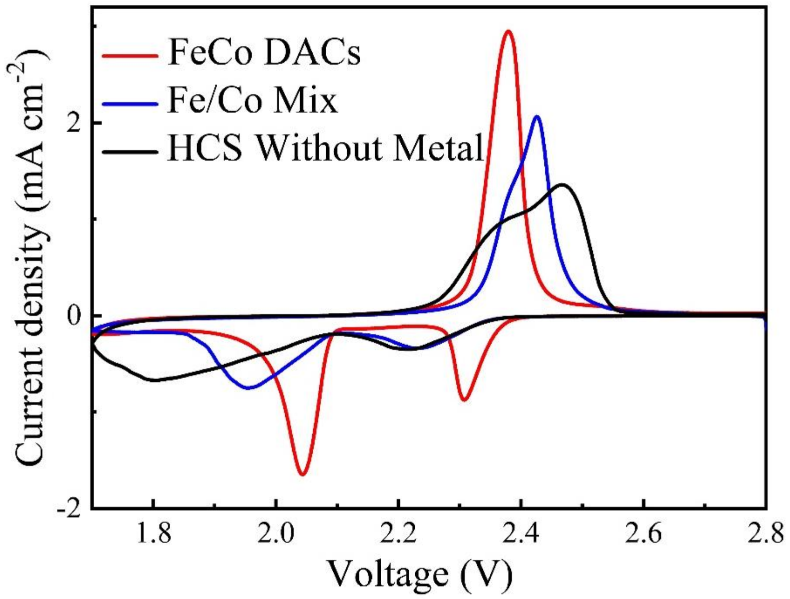

Supplementary Fig. 11 (a) CV curves different cathodes at 0.1 mV s^{-1}.(FeCo DACs/S cathode,Fe/Co mix/S cathode and hollow carbon sphere/S cathode)

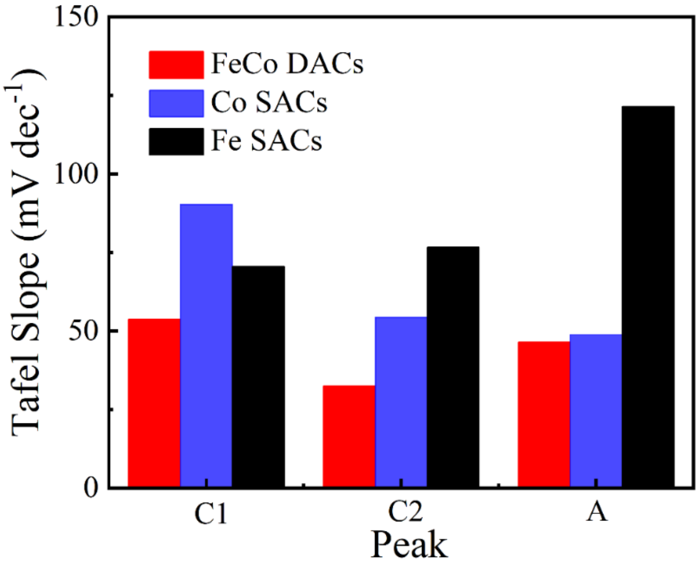

Supplementary Fig. 12 Statistical plot of Tafel slope of fitted curves.

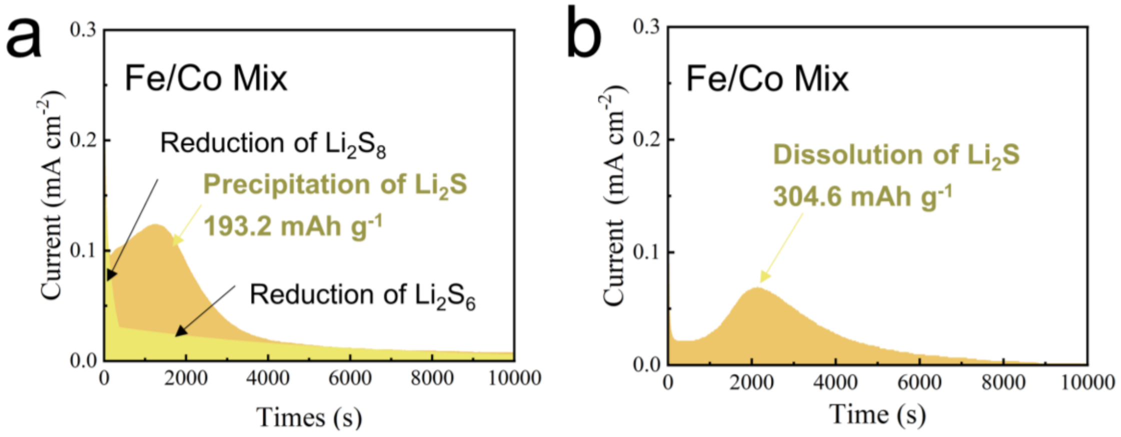

Supplementary Fig. 13 (a)Potentiostatic nucleation curves of Li_2S with Fe SACs and Co SACs mixed. (b)The dissolution profiles of Li_2S.

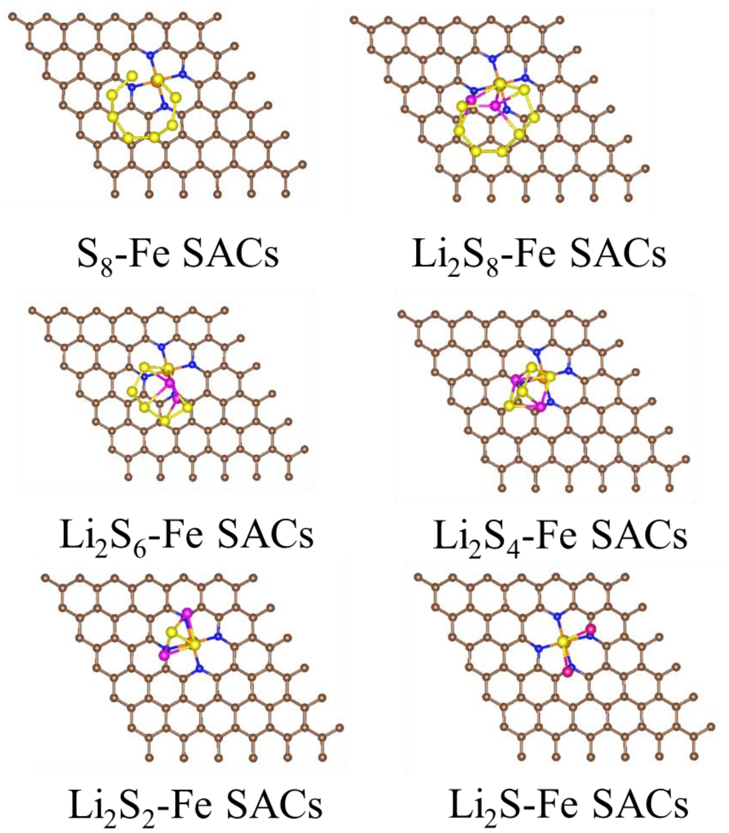

Supplementary Fig. 14 Optimized configurations of sulfur species anchored on Fe SACs.

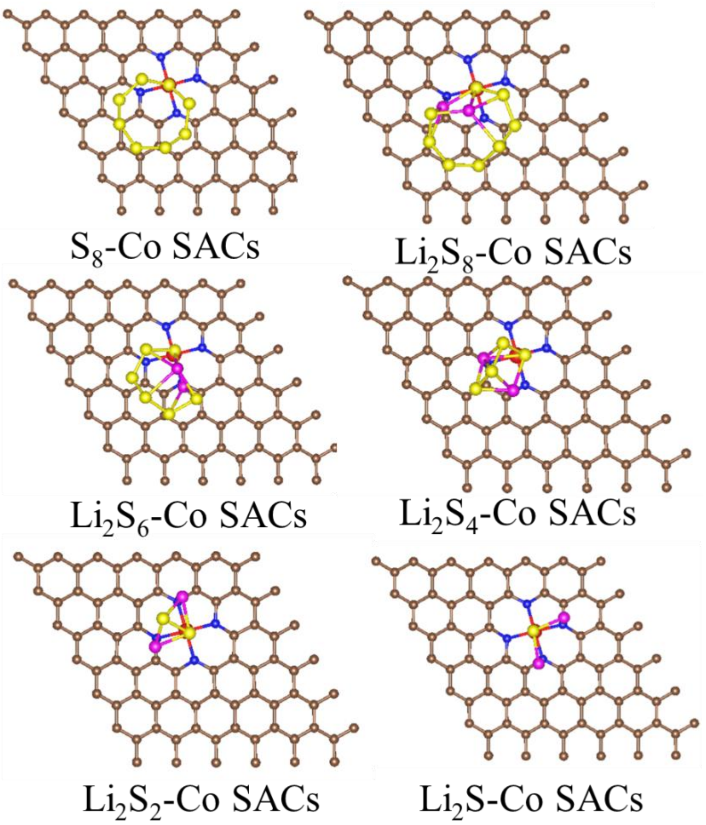

Supplementary Fig. 15 Optimized configurations of sulfur species anchored on Co SACs.

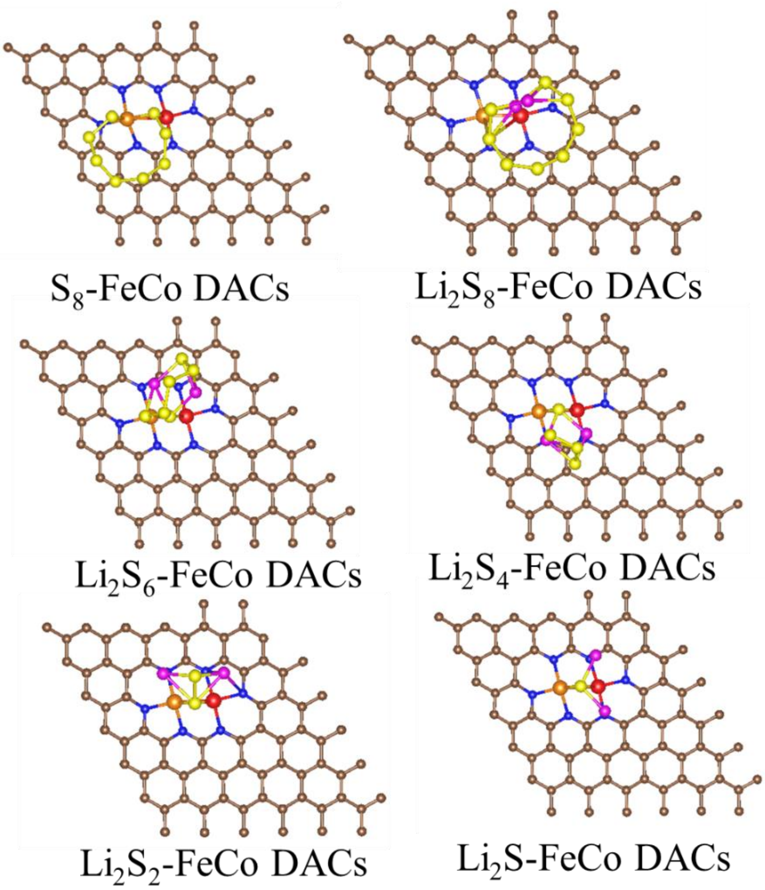

Supplementary Fig. 16 Optimized configurations of sulfur species anchored on Fe-Co DACs.

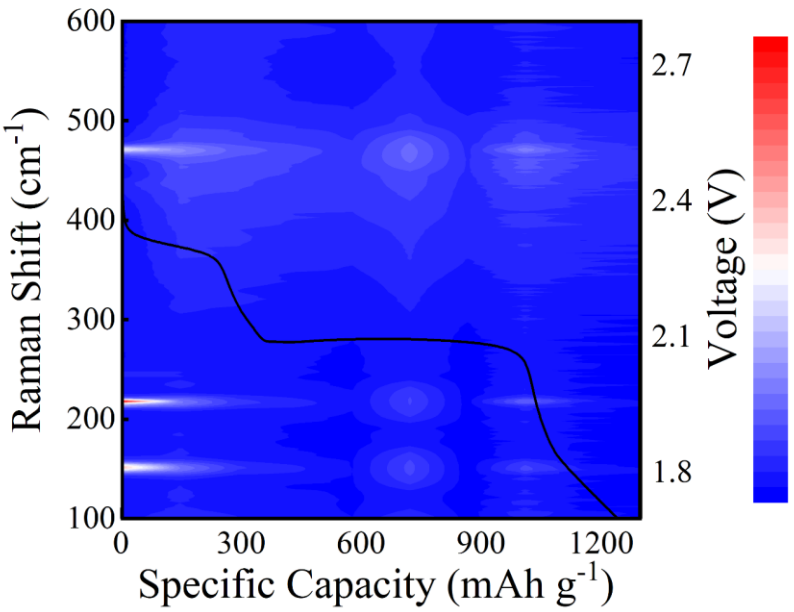

Supplementary Fig. 17 In situ Raman contour plots and corresponding discharging curve of Fe SACs/S cathode.

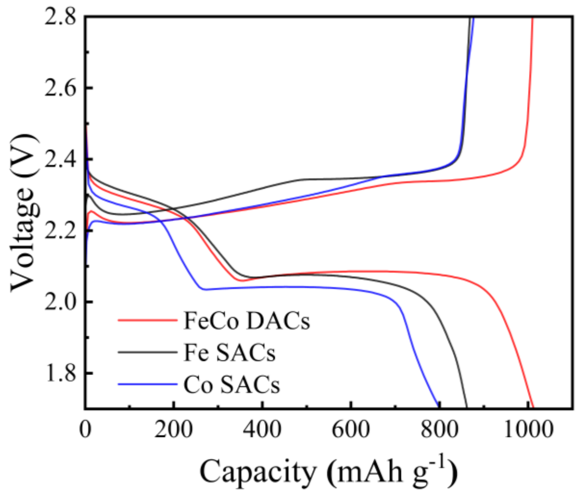

Supplementary Fig. 18 Galvanostatic charge-discharge curves of different cathodes at 1C.

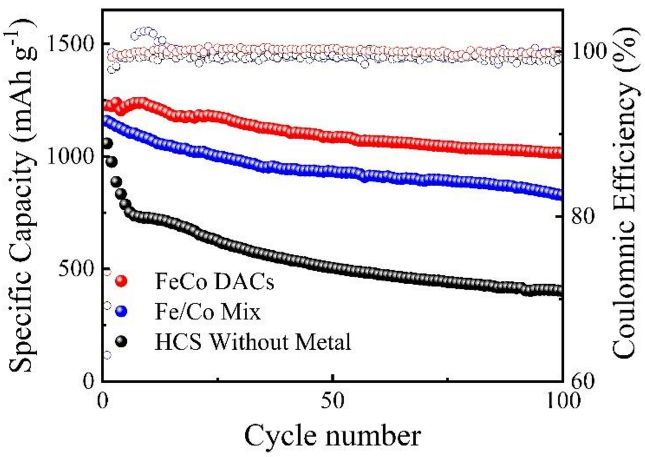

Supplementary Fig. 19 Cycling performance of different cathodes at 0.2 C.

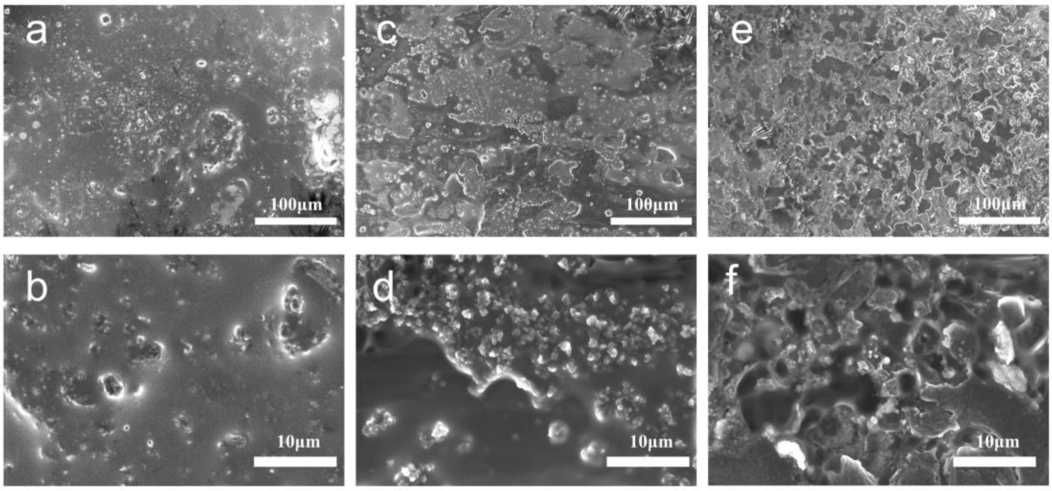

Supplementary Fig. 20 SEM images of Li anodes after 200 cycles under 1 C with (a-b) FeCo DACs, (c-d) Fe SACs, (e-f) Co SACs.

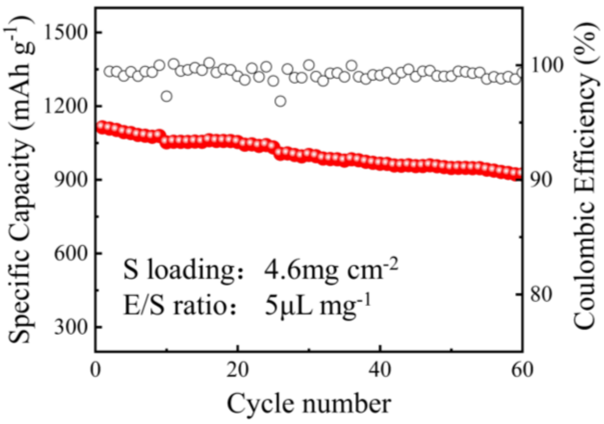

Supplementary Fig. 21 Cycling performance of FeCo DACs/S cell with lean electrolyte at 0.1 C.

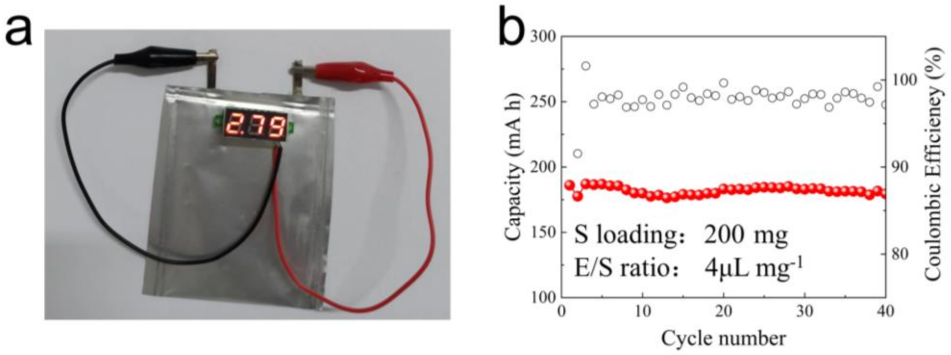

Supplementary Fig. 22 (a) Photograph of Li-S pouch cell (b) Cycling performance of pouch cell at 0.1C.

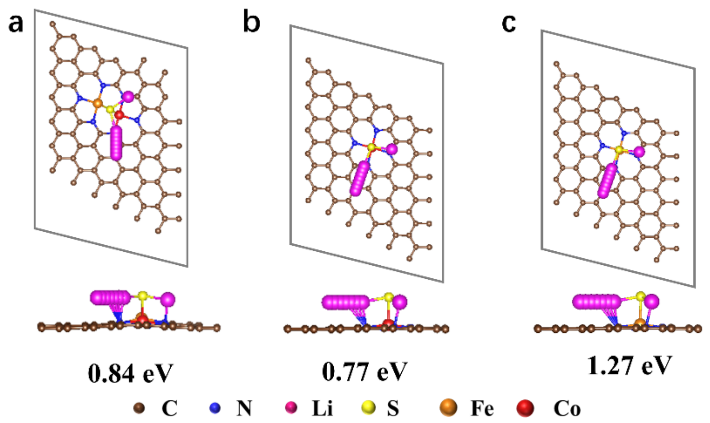

Supplementary Fig. 23 Detailed decomposition path and decomposition barriers for Li_2S on (a) FeCo DACs, (b) Co SACs and (c) Fe SACs.

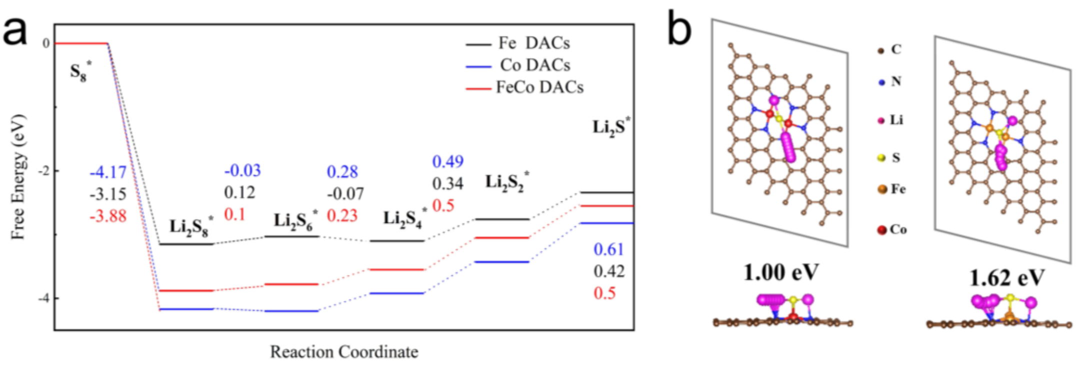

Supplementary Fig. 24 (a) Gibbs free energy of sulfur reduction process on Fe-Co DACs, Co DACs and Fe DACs. (b) Detailed decomposition path and decomposition barriers for Li_2S on Co DACs (left) and Fe DACs (right). (Transition states for elementary reaction steps were determined by a combination of the nudged elastic band (NEB) method and the dimer method. The path between the reactant and product is discretized into a series of structural images; The closest transition state structure image was employed as an initial guess structure for the dimer method.)

Supplementary Table 1. EXAFS fitting parameters at the Co K–edge for various samples

Sample	Shell	CN^a	R(Å)^b	σ^2(Å^2)^c	ΔE_0(eV)^d	R factor
Co foil	Co-Co	12*	2.49±0.01	0.0062±0.0002	7.2±0.3	0.0007
Co_3O_4	Co-O	5.5	1.90±0.03	0.0022±0.0011	8.7±1.0	0.0088
	Co-O-Co1	6.2	2.89±0.01	0.0022±0.0015	6.9±1.4	
	Co-O-Co2	5.4	3.31±0.04	0.0023±0.0019	-2.2±1.0	
CoPc	Co-N	4.1	1.91±0.04	0.0029±0.0009	8.1±2.4	0.0049
	Co-Co	3.6	3.07±0.02	0.0078±0.0031	-9.6±1.9	
**Co sample**	**Co-N1**	**2.0**	**1.88±0.02**	**0.0032±0.0010**	**-8.2±2.5**	**0.0106**
	**Co-N2**	**1.1**	**2.03±0.02**	**0.0064±0.0012**	**7.7±2.0**	
	**Co-Fe**	**0.8**	**2.55±0.01**	**0.0086±0.0065**	**-5.4±1.7**	

^{a}*CN*, coordination number; ^{b}*R*, distance between absorber and backscatter atoms; $^c\sigma^2$, Debye-Waller factor to account for both thermal and structural disorders; $^d\Delta E_0$, inner potential correction; *R* factor indicates the goodness of the fit. S_0^2 was fixed to 0.73, according to the experimental EXAFS fit of Fe foil by fixing CN as the known crystallographic value. A reasonable range of EXAFS fitting parameters: $0.700 < S_0^2 < 1.000$; $CN > 0$; $\sigma^2 > 0$ Å^2; $|\Delta E_0| <$ 10 eV; R factor < 0.02.

Supplementary Table 2. EXAFS fitting parameters at the Fe K–edge for various samples

Sample	Shell	*CN*[a]	*R*(Å)[b]	σ^2(Å^2)[c]	ΔE_0(eV)[d]	*R* factor
Fe foil	Fe-Fe1	8*	2.47±0.07	0.0047±0.0013	5.4±1.7	0.0016
	Fe-Fe2	6*	2.84±0.09	0.0051±0.0022	3.5±3.0	
Fe_2O_3	Fe-O	6.1	1.98±0.03	0.0075±0.0022	7.2±1.7	0.0067
	Fe-O-Fe1	6.7	2.98±0.01	0.0067±0.0013	8.5±1.5	
	Fe-O-Fe2	4.3	3.65±0.01	0.0017±0.0005	2.6±1.6	
FePc	Fe-N	4.2	1.95±0.01	0.0060±0.0040	5.7±2.0	0.0127
	Fe-Fe	4.0	3.09±0.04	0.0076±0.0069	7.2±1.8	
**Fe sample**	**Fe-N1**	**2.1**	**1.98±0.01**	**0.0039±0.0014**	**7.3±2.5**	**0.0101**
	**Fe-N2**	**1.0**	**2.06±0.02**	**0.0027±0.0020**	**8.4±2.2**	
	**Fe-Co**	**0.7**	**2.51±0.04**	**0.0054±0.0031**	**6.9±1.4**	

[a]*CN*, coordination number; [b]*R*, distance between absorber and backscatter atoms; [c]σ^2, Debye-Waller factor to account for both thermal and structural disorders; [d]ΔE_0, inner potential correction; *R* factor indicates the goodness of the fit. S_0^2 was fixed to 0.75, according to the experimental EXAFS fit of Fe foil by fixing CN as the known crystallographic value. A reasonable range of EXAFS fitting parameters: $0.700 < S_0^2 < 1.000$; $CN > 0$; $\sigma^2 > 0$ Å^2; $|\Delta E_0| < 10$ eV; R factor < 0.02.

Supplementary Table 3. EXAFS data fitting results of Fe-Fe.

Sample	Shell	CN^a	R(Å)^b	σ^2(Å^2)^c	ΔE_0(eV)^d	R factor
**Fe sample-1**	**Fe-N1**	**2.1**	**1.98**	**0.0041**	**7.5**	**0.0125**
	**Fe-N2**	**1.0**	**2.06**	**0.0025**	**8.5**	
	**Fe-Fe**	**0.7**	**2.50**	**0.0068**	**8.1**	

Supplementary Table 4. EXAFS data fitting results of Co-Co.

Sample	Shell	CN^a	R(Å)^b	σ^2(Å^2)^c	ΔE_0(eV)^d	R factor
**Co sample-1**	**Co-N1**	**2.0**	**1.88**	**0.0031**	**-8.1**	**0.0122**
	**Co-N2**	**1.1**	**2.03**	**0.0063**	**7.6**	
	**Co-Co**	**0.8**	**2.57**	**0.0071**	**-6.1**	

Supplementary Table 5. Elemental composition for the catalysts (ICP).

Sample	Iron (wt.%)	Cobalt (wt.%)
**Fe SACs**	2.13	/
**Co SACs**	/	2.15
**FeCo DACs**	1.08	1.03
